# Mechanical Behavior and Mix Design Optimization of Hybrid Mudstone–Sandstone Coal Gangue Aggregate Concrete

**DOI:** 10.3390/ma19214615

**Published:** 2026-10-30

**Authors:** Fengzhen He, Guichen Li, Luigi Biolzi, Jinghua Li, Juli Liang, Changlun Sun

**Affiliations:** 1 Joint National-Local Engineering Research Centre for Safe and Precise Coal Mining, Anhui University of Science and Technology, Huainan 232001, China; 2 School of Mines, China University of Mining and Technology, Xuzhou 221116, China; 3 Department of Architecture, Built Environment and Construction Engineering, Politecnico di Milano, Piazza Leonardo Da Vinci 32, 20133 Milan, Italy; 4 Changsha Institute of Mining Research Co., Ltd., Changsha 410012, China; 5 School of Mines, Guizhou Institute of Technology, Guiyang 550003, China

**Keywords:** gangue lithology, gangue-based concrete, mechanical properties, mix optimization

## Abstract

Recycling crushed coal gangue as concrete aggregate enables the clean, low-carbon, and sustainable disposal of coal gangue. This paper systematically investigates how gangue lithology and aggregate grading parameters jointly affect the mechanical properties and mixture design of coal gangue concrete. Test results show that higher grading parameters of coal gangue aggregate improve concrete workability. For grading parameters under 0.4, compressive strength fluctuates with water–cement ratio, whereas it decreases monotonically with increasing water–cement ratio when grading parameter exceeds 0.4. The optimal mixture is determined as water–cement ratio 0.6 and grading parameter 0.6. Mudstone gangue drastically modifies the compressive stress–strain behavior of gangue concrete. Compressive strength and elastic modulus drop by 28.3% and 22.4%, respectively, as mudstone content increases from 0% to 75%, with sharp mechanical degradation observed beyond the 75% threshold. As mudstone proportion rises from 25% to 100%, the dominant failure mode shifts from interface debonding and matrix cracking to a composite failure involving interface debonding, aggregate fracture and matrix rupture. For on-site construction, supplementary non-argillaceous gangue is recommended for mixtures with a mudstone content over 75% to maintain acceptable mechanical performance of coal gangue aggregate concrete.

## 1. Introduction

**Figure 1:**
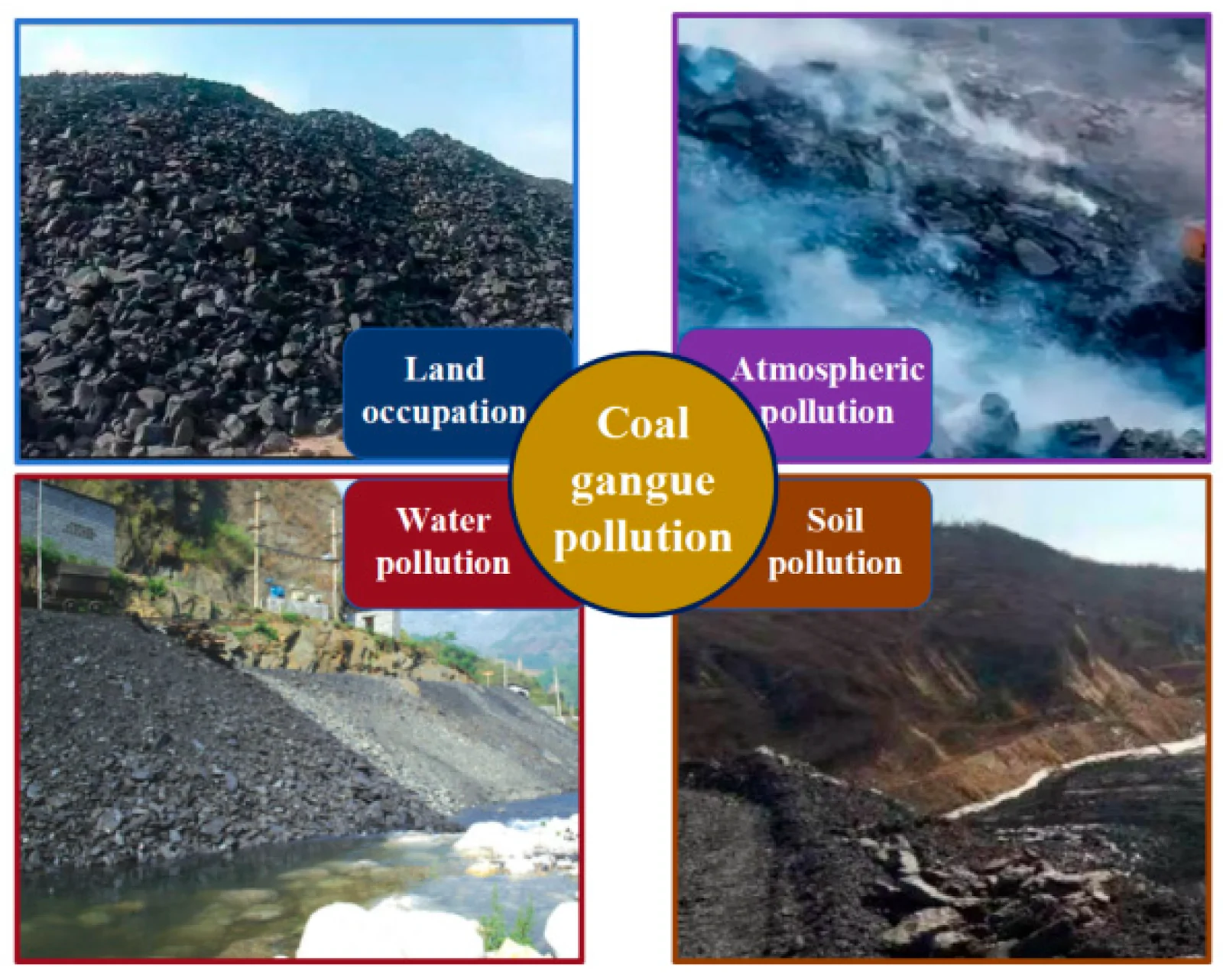
Environmental and aquatic pollution risks caused by coal gangue.

**Figure 2:**
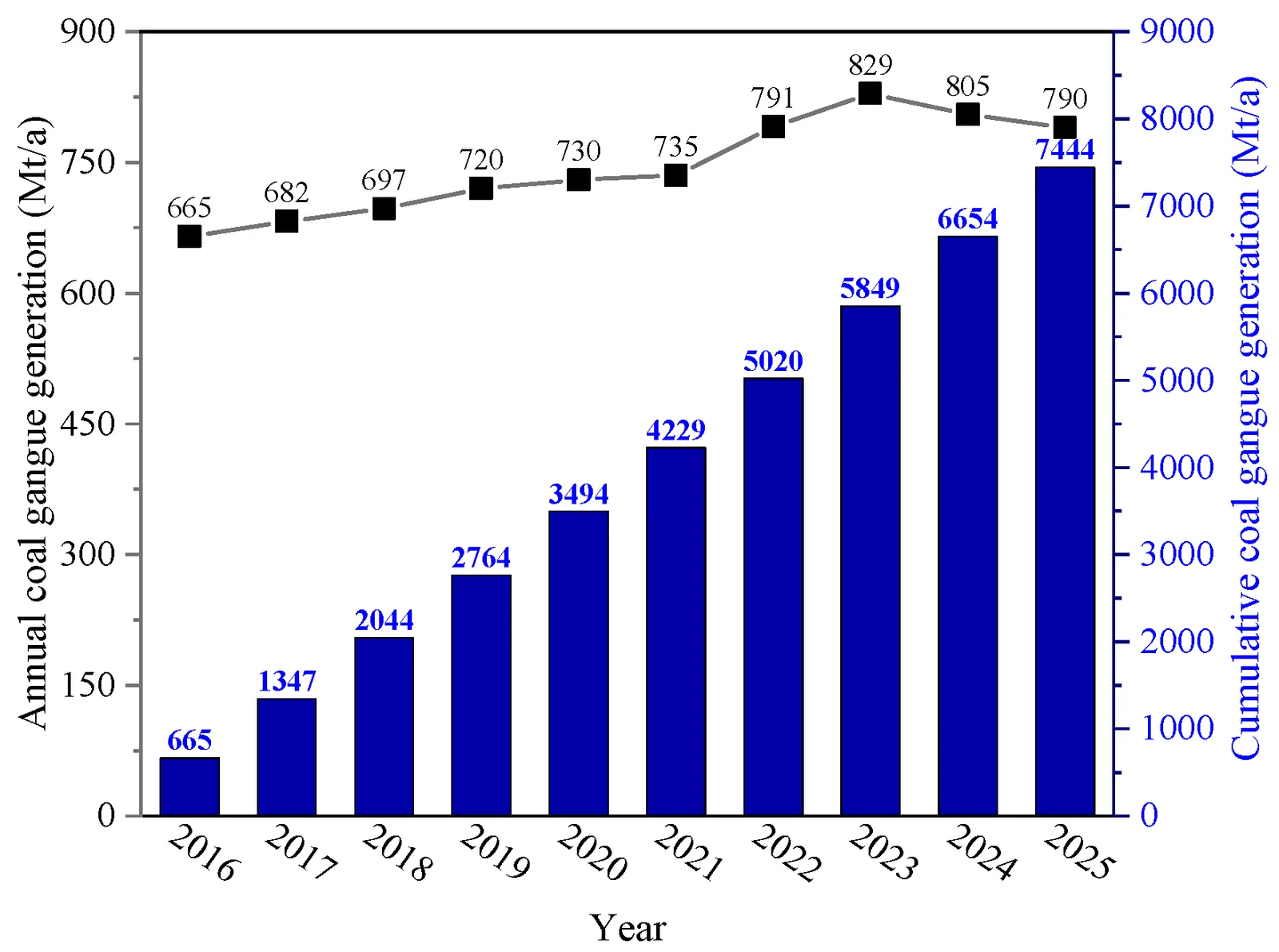
Coal gangue production in China from 2016 to 2025.

Coal gangue, an inevitable solid waste by-product generated during coal mining and washing processes, poses considerable challenges due to its extensive accumulation [1,2]. This accumulation not only consumes valuable land resources but also entails potential environmental and aquatic pollution risks (Figure 1), which may trigger catastrophic incidents, such as spontaneous combustion and collapse [3,4]. As the world’s largest coal-producing country, China generates coal gangue equivalent to 10–15% of its total raw coal output each year, with the annual production volume ranging from 660 to 890 million tonnes and maintaining a sustained upward trend (Figure 2) [2,5]. By 2025, the cumulative stockpile of coal gangue in China had exceeded 8.5 billion tons, making it one of the countries with the highest emissions of industrial solid waste globally [6,7]. This massive accumulation not only exacerbates the complexity of solid waste management but also imposes more stringent requirements for the resourceful and environmentally friendly utilization of coal gangue [8,9,10].

An innovative and effective strategy for the clean utilization of coal gangue involves crushing it and subsequently applying it as concrete aggregates. This approach not only reduces reliance on natural aggregates, lowering the production cost of concrete, but also facilitates the resource-oriented utilization of coal gangue [2,11,12]. Gangue concrete is extensively utilized as mine backfill, mining shotcrete and pavement base materials. Meanwhile, incorporating coal gangue as concrete aggregate lowers carbon emissions, which serves as a promising pathway for developing low-carbon concrete.

In recent years, extensive studies [13,14,15,16] have been conducted on the physical and mechanical properties of coal gangue, as well as the mechanical performance of coal gangue-based concrete. These studies have demonstrated that crushed coal gangue can serve as a viable alternative to natural aggregates in concrete production. Such findings provide a fundamental basis for the widespread application of coal gangue in engineering construction. However, owing to the highly complex and variable composition and physicochemical properties of coal gangue, the mechanical characteristics of coal gangue-based concrete can vary significantly depending on the type and content of gangue used [17,18]. Most existing research on coal gangue aggregate concrete has paid little attention to the effect of coal gangue lithology, and the performance impact of coal gangue with different lithologies remains poorly understood. Conventionally, coal gangue of different lithologies (such as mudstone and sandstone) is mixed and processed without differentiation, which makes it difficult to control experimental variables and ensure consistent quality of coal gangue aggregate concrete. Therefore, understanding the influence of coal gangue lithology on the performance of coal gangue-based concrete is essential for optimizing its mix design. This study systematically investigates the effect of coal gangue derived from both mudstone and sandstone lithologies on the mechanical performance of coal gangue aggregate concrete.

To date, most prior studies have predominantly focused on the mechanical behavior and durability of concrete incorporating unclassified coal gangue, whereas few investigations have addressed the mix proportion optimization of gangue-based concrete that simultaneously accounts for the lithological characteristics and gradation parameters of gangue aggregates, such as aggregate particle size, size distribution, and proportions of different particle size fractions. In practical engineering, coal gangue generally comprises multiple lithological fractions with disparate mineral compositions [19,20]. Economical and efficient technologies for sorting gangue by lithology are currently lacking for large-scale industrial deployment. Accordingly, gangue-based concrete in practical engineering invariably contains blended gangue lithologies including mudstone, sandstone and granite, which inevitably induces fluctuations in the mechanical performance of the resultant composites [21,22]. Accurate clarification of how blended gangue assemblages alter concrete performance is therefore essential for on-site quality control. Nevertheless, there remains a notable research gap regarding the coupled effects of mixed gangue lithologies on the macro-properties of gangue-based concrete. The existing literature fails to systematically differentiate samples of varying lithologies, and correlation models among particle size distribution, mineral composition and mechanical characteristics remain lacking. Mudstone and sandstone represent the two most dominant gangue fractions produced in coal mining activities. Excess mudstone fractions exert severe detrimental impacts on the comprehensive performance of gangue concrete [5]. Meanwhile, the complexity of water-rock interaction mechanisms of mudstone, such as water absorption and expansion of clay minerals, further increases the difficulty of systematic research of the mechanical performance of the coal gangue aggregate concrete [23,24]. In this context, quantifying the influence of mudstone content and particle size on the mechanical responses of gangue-based concrete carries great theoretical and engineering significance.

This paper systematically investigated the mechanical performance and mix proportion optimization of gangue-based concrete incorporated with mudstone and sandstone gangue. First, the material composition and physical structural characteristics of coal gangue with different lithologies were analyzed to reveal its potential influence mechanisms on the macroscopic performance of concrete. Second, the effects of aggregate grading parameters on the mechanical properties of coal gangue concrete were comprehensively explored. Finally, through macroscopic physical and mechanical tests combined with microstructural characterization, the mechanical behaviors of gangue-based concrete with varying mudstone-to-sandstone mass ratios were determined, clarifying the influence law of mixed lithological gangue aggregates on the mechanical performance of concrete. The threshold of the mudstone coal gangue content for the coal gangue aggregate concrete was determined. The research findings can provide a solid theoretical foundation and practical technical reference for the mix design and performance optimization of mudstone-containing gangue concrete, particularly for the proportioning design of composite coal gangue aggregates in engineering practice.

## 2. Materials and Testing Method

### 2.1. Materials

The types of gangue lithologies analyzed were mudstone and sandstone. Both gangue specimens were obtained from the rock driving roadway of Coal Mine, and the cement used was Portland Cement CEM II/A-LL42.5R. The gangue collected from the site was processed into cylindrical specimens (diameter: 50 mm, height: 100 mm) and disk-shaped specimens (diameter: 50 mm, thickness: 25 mm) for testing the uniaxial compressive strength and splitting tensile strength. The tests were conducted using a universal servo-controlled testing machine (Changchun Equipment and Instrument Factory, Changchun, China).

**Figure 3:**
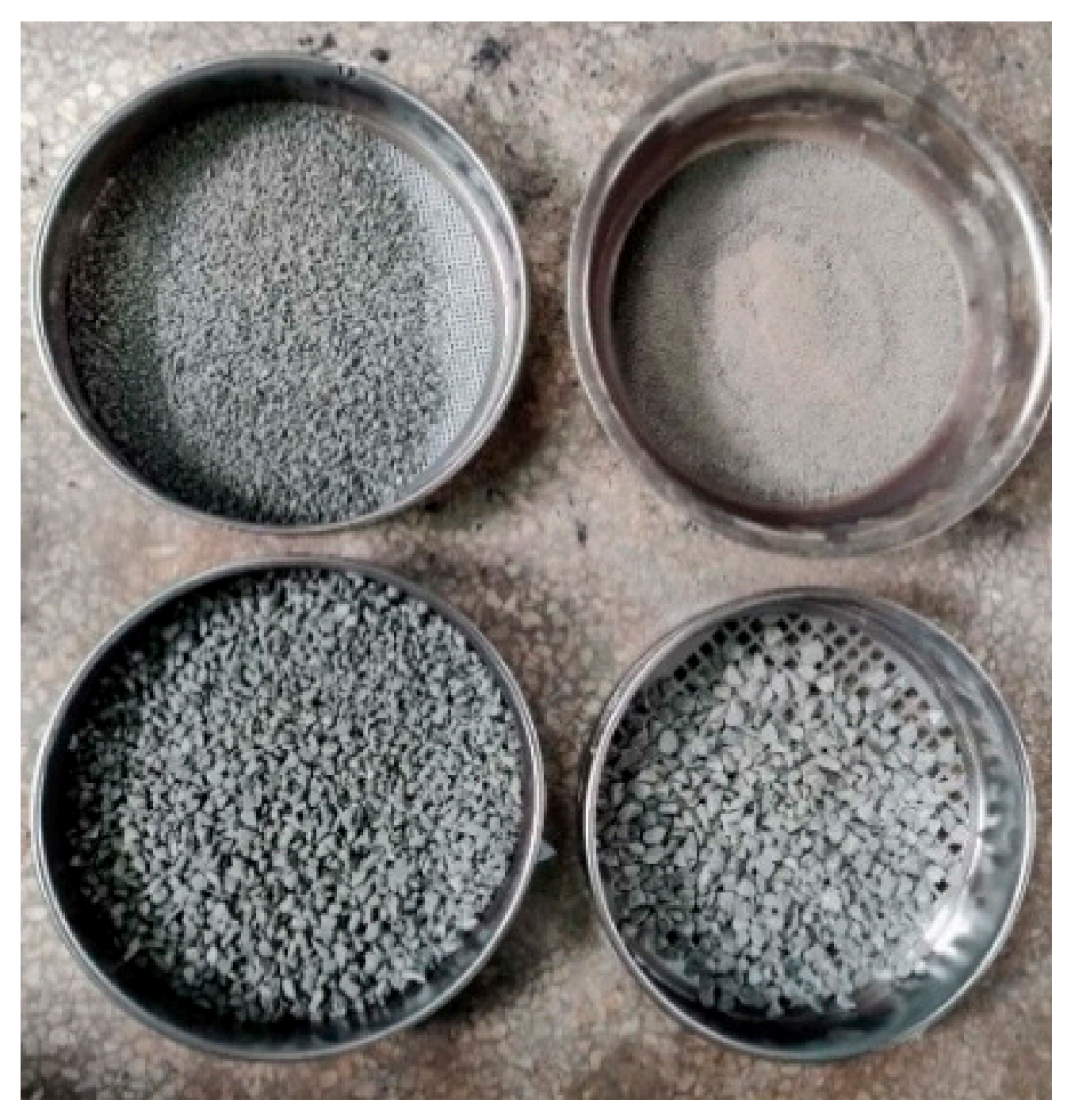
Crushed gangue aggregate with different particle sizes.

**Figure 4:**
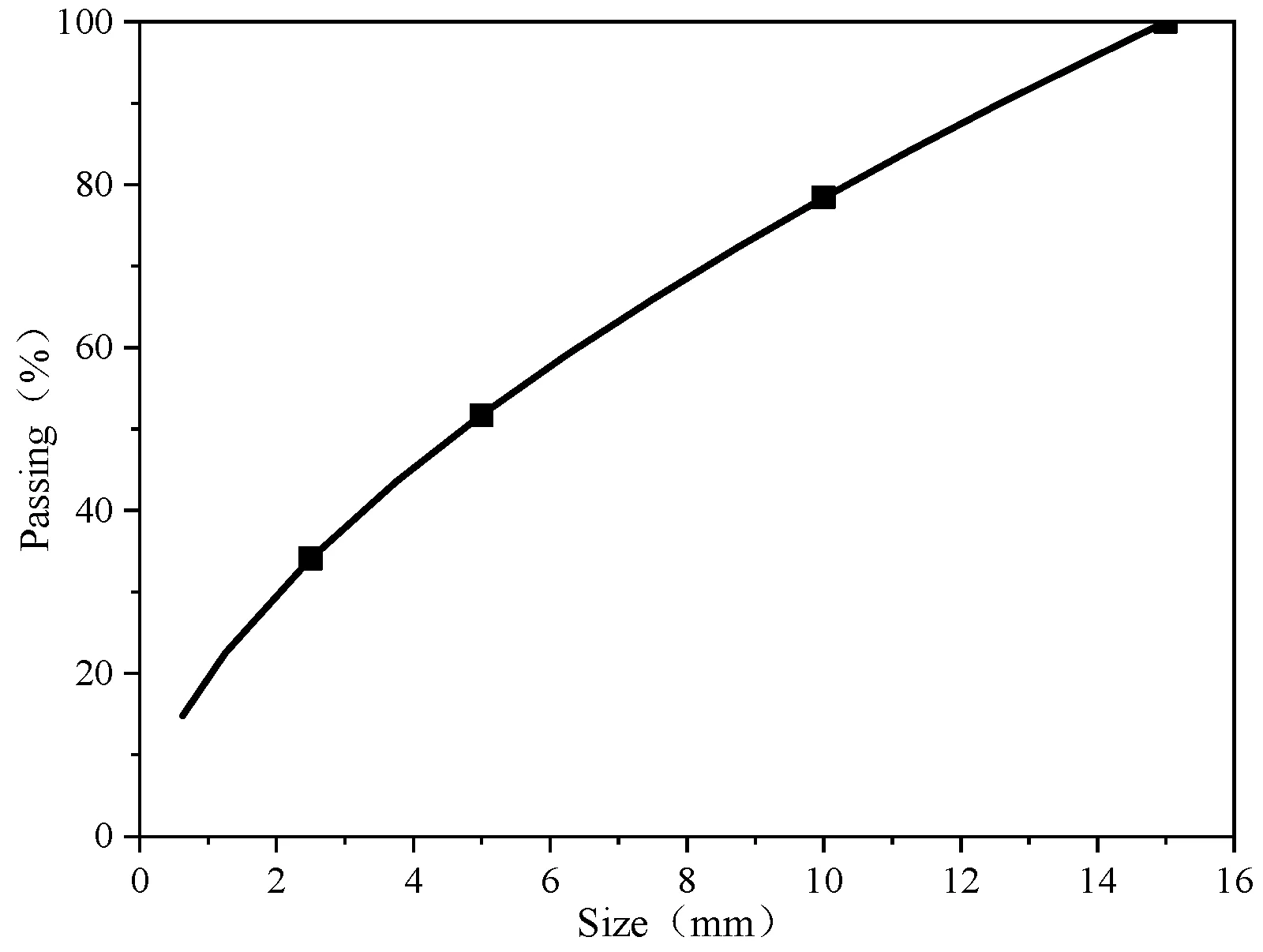
Grading curve of gangue aggregate.

According to Construction Standard for Coal Mine Roadway Engineering (GB/T 50511-2022) [25], the aggregate particle size of shotcrete used in coal mines shall not be greater than 15 mm. Bulk gangue collected from the site was crushed using a jaw crusher to a particle size range from 0 to 15 mm. Square-mesh sieves were employed to separate the crushed rock into four different particle size fractions: 0 to 2.5 mm, 2.5 mm to 5.0 mm, 5.0 mm to 10.0 mm, and 10.0 mm to 15.0 mm, serving as gangue aggregates (see Figure 3). Limestone aggregates with a particle size of 5.0 to 15.0 mm were selected as the coarse aggregates for the control group (Con.G), while coarse-to-medium sand with a fineness modulus greater than 2.5 was used as the fine aggregates for the control group. In this paper, the grading of coarse and fine aggregates in the gangue concrete was designed based on the Talbot grading theory, with a grading coefficient of 0.6. The grading curve is presented in Figure 4.

Since the particle size distribution of crushed coal gangue conforms to the Talbot gradation curve [26] (Equation (1)), this study employed the Talbot gradation theory to analyze the influence of gradation on the workability of concrete. For mineral mixtures, *n* should be within the range of 0.3 to 0.7 [27,28]. Five gradation schemes were designed with n values of 0.3, 0.4, 0.5, 0.6, and 0.7, respectively. The water–cement ratio (R) for ordinary concrete generally ranges from 0.4 to 0.8. The analysis considered water–cement ratios of 0.5, 0.55, 0.6, 0.65, and 0.7. Based on the selected gradation parameters and water–cement ratios, 25 test groups were designed, as shown in detail in Table 1 and Table 2. The control group’s aggregate is crushed limestone and river sand.

(1) $$P=100{\left(\frac{d_{i}}{D}\right)}^{n}$$

where *P* is the percentage of aggregate particles with a diameter smaller than *di*, *di* is the diameter of the aggregate particles (mm), *D* is the maximum diameter of the aggregate (mm), and *n* is the gradation parameter of the aggregate.

### 2.2. Mix Design and Specimen Casting

To determine the optimal mix proportions of coal gangue-based concrete, 25 different mix formulations (Table 1 and Table 2) were evaluated to assess the effects of aggregate gradation and water–cement ratio on workability and uniaxial compressive strength. Additionally, six groups of gangue-based concrete containing gangue aggregate with different lithologies were designed to investigate the effects of gangue lithology and combinations of different lithological gangues on the mechanical properties of gangue-based concrete. The specific mix proportions for the different sets are detailed in Table 3. The Mix ID including the type of gangue and its proportion is adopted. For example, MG0.5SG0.5 represents 50% mudstone and 50% sandstone gangue in this mixture. MG1 and SG1 are mixtures including single lithology gangue. Con.G serves as a control group, representing a standard concrete specimens with limestone as the coarse aggregate and river sand as the fine aggregate. MG0.25SG0.75, MG0.5SG0.5, and MG0.75SG0.25 are mixed groups containing two different types of coal gangues.

According to the Standard for testing methods of physical and mechanical properties of concrete (GB/T 50081-2019) [29], square specimens with a side length of 50 mm were cast and cured in molds for one day. Afterward, the specimens were demolded and placed in a curing chamber at a temperature of 20 ± 2 °C and humidity of 90 ± 3% until the testing time.

### 2.3. Testing Methods

Mineral composition testing of gangue was conducted using a D8ADVANCE X-ray diffractometer (XRD) (Bruker AXS GmbH, Karlsruhe, Germany). Water immersion tests were carried out on coal gangue to assess the stability of different lithological coal gangue upon contact with water. Uniaxial compression tests and splitting tests were conducted on gangue specimens according to the international rock mechanics testing standard [30]. Cylinder specimens with dimensions of 50 mm in diameter and 100 mm in height were utilized for compression testing. Disc specimens, featuring dimensions of 50 mm in diameter and an unspecified thickness, were employed for splitting tests. Loading was applied in a displacement-controlled method at a rate of 0.02 mm/s. According to the Standard for quality control of concrete (GB50164-2011) [31], the workability of different gangue-based concretes was tested using the slump test. Uniaxial compression tests were performed on gangue-based concrete specimens at ages of 1, 7, 14, and 28 days. For each mixing group and its corresponding testing age, a minimum of three specimens underwent testing, and the compressive strength was determined by calculating the average value obtained from these tests.

## 3. Physical and Mechanical Properties of Coal Gangue

### 3.1. XRD and XRF Analysis

**Figure 5:**
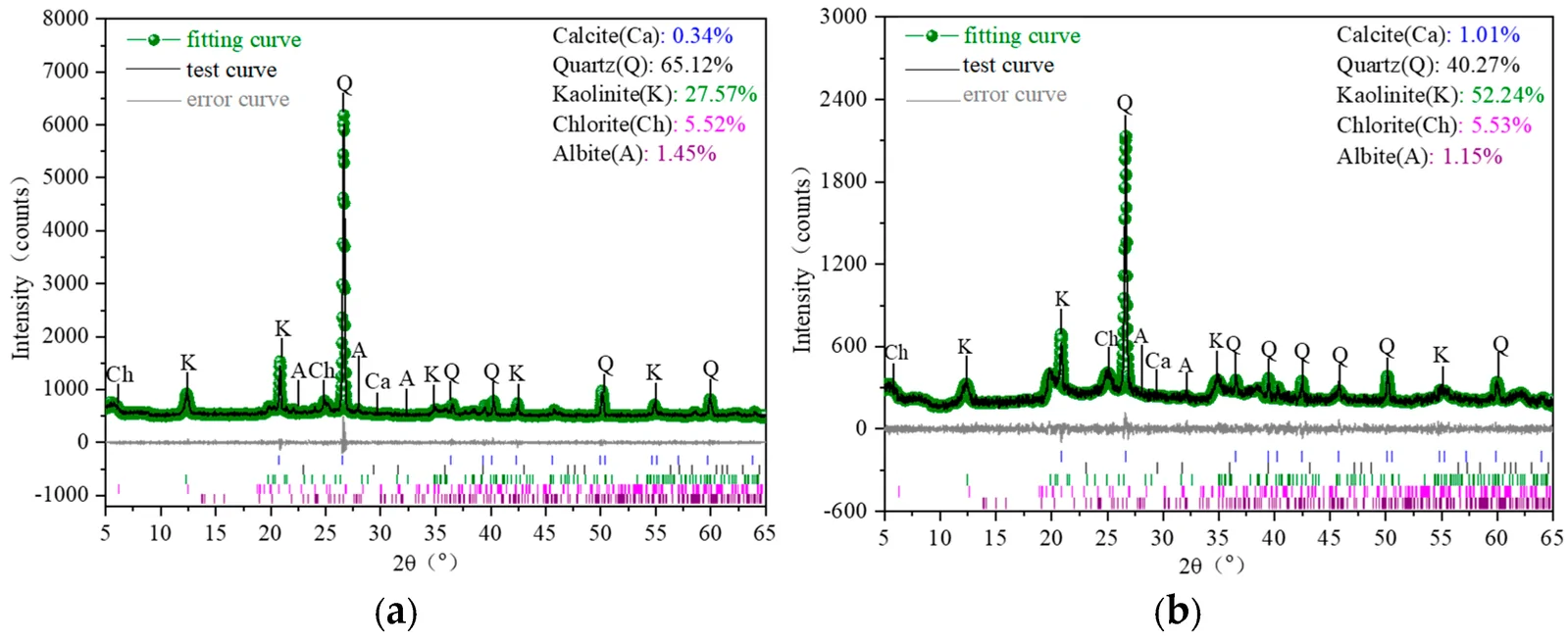
Mineral composition of (**a**) mudstone gangue, and (**b**) sandstone gangue.

The analysis of the chemical composition and mineral composition of coal gangue provides fundamental data for the optimal design and performance adjustment of gangue-based concrete [32,33]. XRD analysis enables the acquisition of the phases and relative content of minerals in coal gangue specimens. The XRD test covers a diffraction angle range of 5° to 65° with a diffraction speed of 2°/min. The XRD test results for the two types of coal gangue are shown in Figure 5.

The main mineral components of mudstone gangue are kaolinite and quartz, with respective contents of 52.24% and 40.27%. The contents of clinozoisite, potassium feldspar, and calcite are 5.53%, 1.15%, and 1.01%, respectively. In sandstone gangue, quartz has the highest proportion, accounting for 65.12%, followed by kaolinite at 27.57%. The contents of clinozoisite, albite, and calcite are 5.52%, 1.45%, and 0.34%, respectively. In terms of mineral composition, mudstone gangue and sandstone gangue have the same mineral components, but the content of clay minerals such as kaolinite in mudstone gangue is twice that of sandstone gangue. The difference in clay mineral content is one of the main reasons for the difference in mechanical properties between mudstone gangue and sandstone gangue.

### 3.2. Mechanical Properties of Coal Gangue

**Figure 6:**
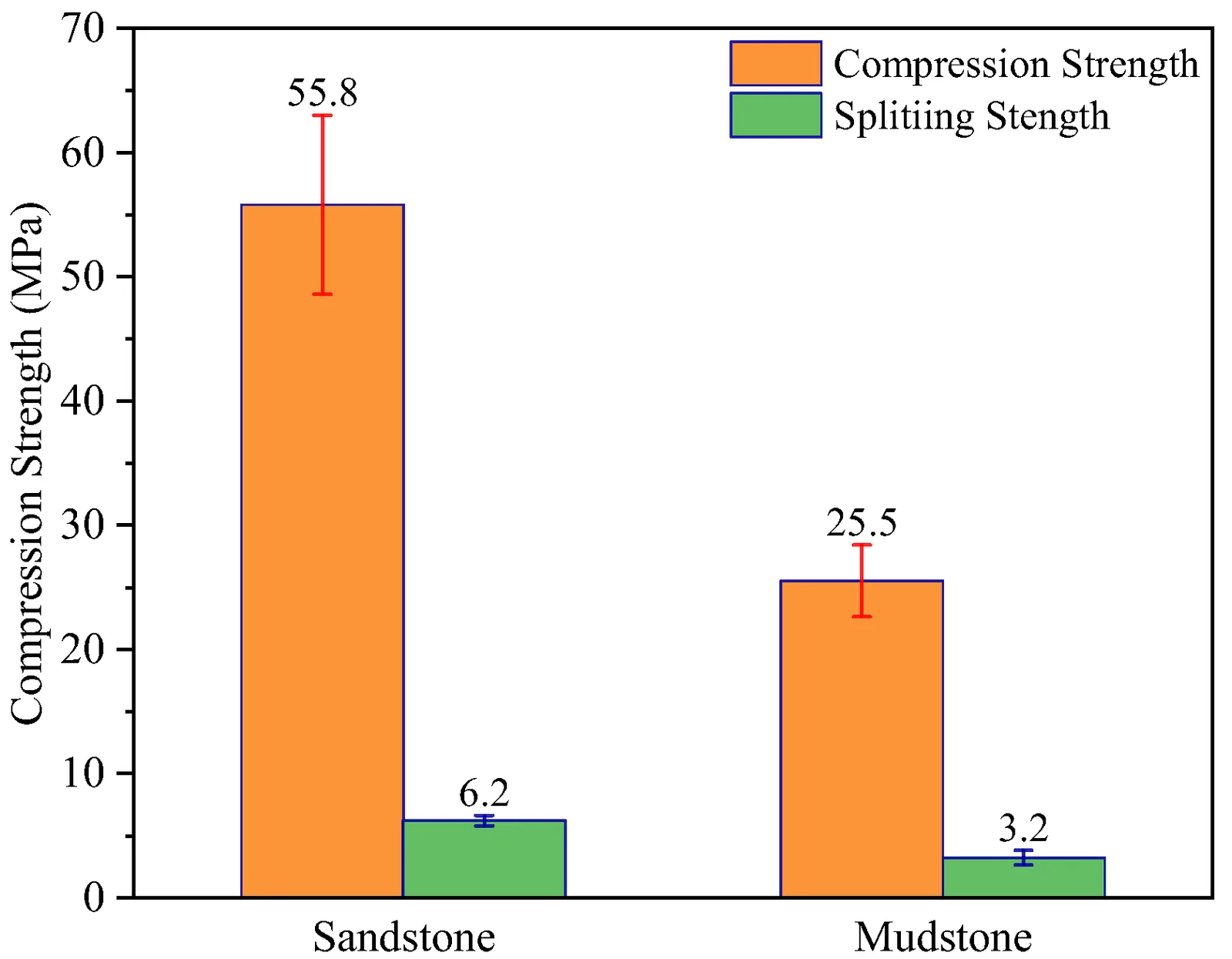
Uniaxial compressive strength and splitting strength of gangue.

Figure 6 shows the uniaxial compressive strength and splitting strength of gangue. For concrete, aggregate serves as one of the primary load-bearing structures within it, and its mechanical strength holds a crucial influence on the mechanical properties of concrete [34,35]. The uniaxial compressive strength and splitting strength of sandstone gangue are 55.8 MPa and 6.2 MPa, respectively, while those of mudstone gangue are 25.5 MPa and 3.2 MPa. This difference is primarily attributed to the presence of numerous natural micro-cracks and pores in mudstone gangue. These original microscopic defects propagate under external loads, ultimately causing cracking of mudstone gangue under relatively low loads. Consequently, mudstone-containing gangue concrete exhibits a high susceptibility to coarse aggregate rupture upon external loading, thereby drastically reducing its load-carrying performance.

## 4. Optimization of Gangue-Based Concrete Mix Proportion

**Figure 7:**
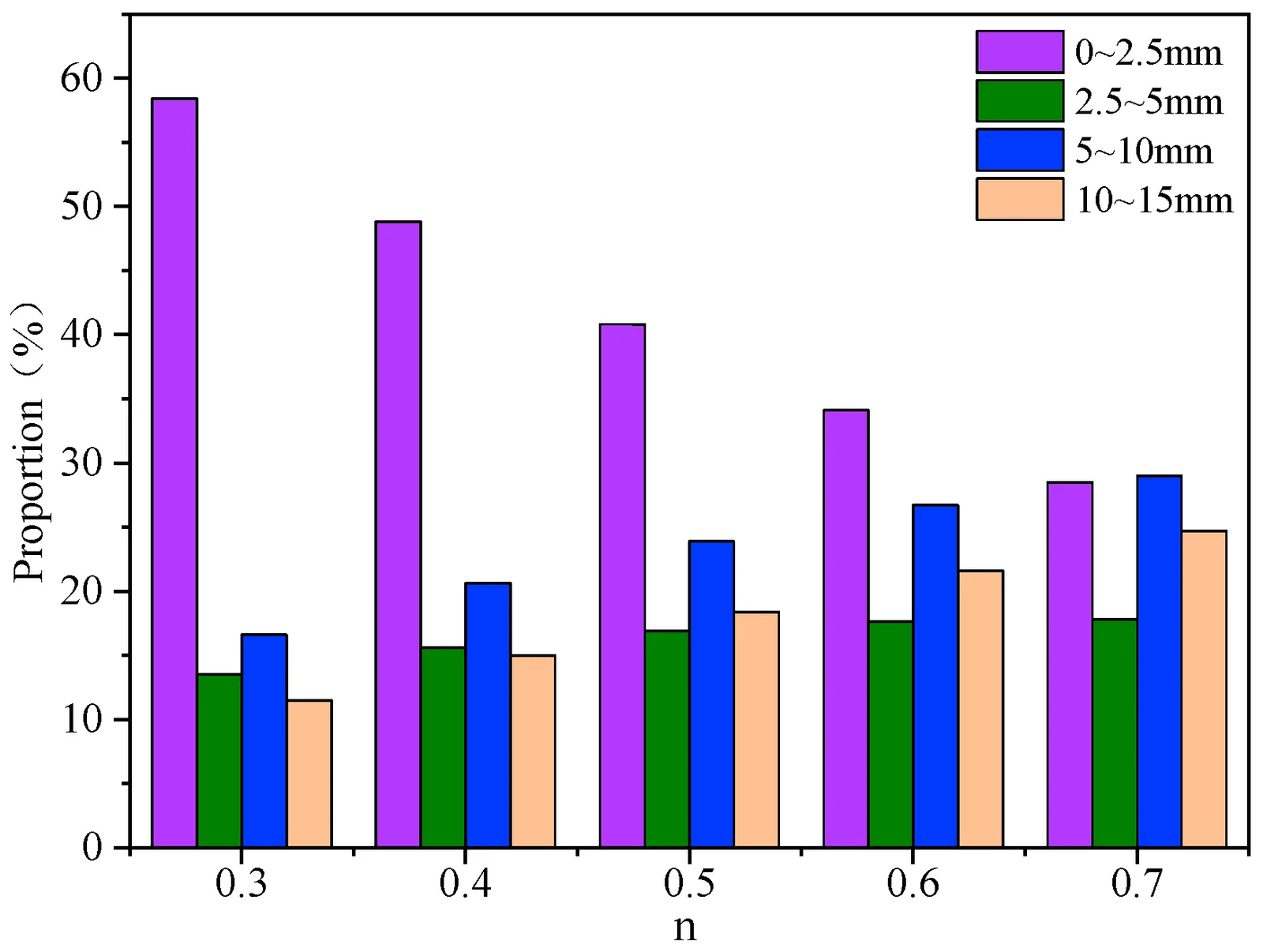
The proportion of aggregate within each particle size range for different grading parameters.

The gangue aggregate gradation and water–cement ratio are two key parameters that affect the mechanical properties of gangue concrete. A reasonable aggregate gradation and water–cement ratio can enable the concrete to form a dense internal structure while possessing good workability and fluidity. The weight proportion of sandstone aggregate particles of different sizes under different gradation parameters is shown in Figure 7.

### 4.1. Workability

**Figure 8:**
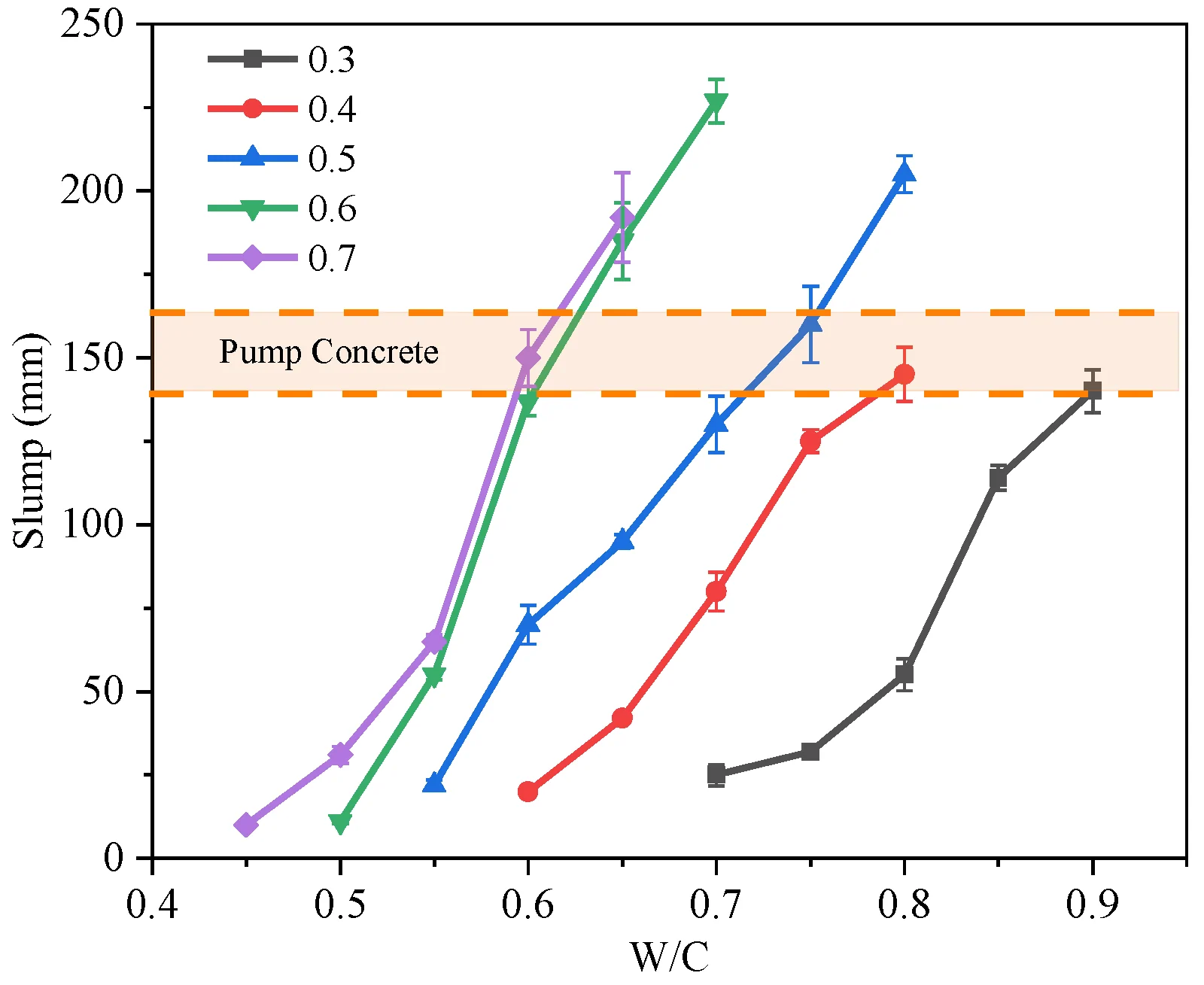
Slump-W/C curve of gangue-based concrete with different grading parameters.

Figure 8 presents the variation curves of the slump of gangue-based concrete with different grading conditions and water–cement ratios. The slump of gangue-based concrete with different grading parameters increases with the water–cement ratio. The higher water–cement ratio leads to more free water being adsorbed on the surfaces of aggregates, thereby reducing the friction between the aggregates and hydration products, which in turn enhances the fluidity of the concrete. For specimens with identical water–cement ratios, the slump value of gangue concrete increases with growing aggregate grading parameter. A dramatic reduction in slump can be observed as the grading parameter n declines from 0.6 to 0.5. For the water–cement ratio of 0.6, the slump decreases from 137 mm to 70 mm when the grading parameter decreases from 0.6 to 0.5, representing a 48.9% decrease. It is primarily related to the specific surface area of aggregates. As shown in Figure 7, the proportion of aggregates with smaller particle sizes decreases as the grading parameter increases. Smaller aggregates possess a larger specific surface area, necessitating the adsorption of more free water on their surfaces to reduce the friction between aggregates and hydration products. Therefore, for grading parameter 0.3, gangue-based concrete tends to agglomerate during mixing when the water–cement ratio is under 0.7. However, when the grading parameter increases from 0.6 to 0.7, no significant change in the slump appears.

According to Standard for Acceptance of Construction Quality of Concrete Structure Engineering (GB 50204-2015) [36], the slump of pumped concrete should be between 140 and 160 mm. For the three types of gangue-based concrete with grading parameters of 0.4, 0.5, and 0.6, the water–cement ratio ranges that meet the slump requirements for pumped concrete are 0.8–0.9, 0.72–0.77, and 0.6–0.65, respectively. Overall, the water–cement ratios that satisfy the fluidity of pumped concrete with different grading parameters fall within a relatively narrow range. For the grading parameters of 0.5, 0.6, and 0.7, good fluidity of gangue concrete can be obtained at lower water–cement ratios, meeting the requirements for pumping.

### 4.2. Compressive Strength of Gangue-Based Concretes with Different Proportions

**Figure 9:**
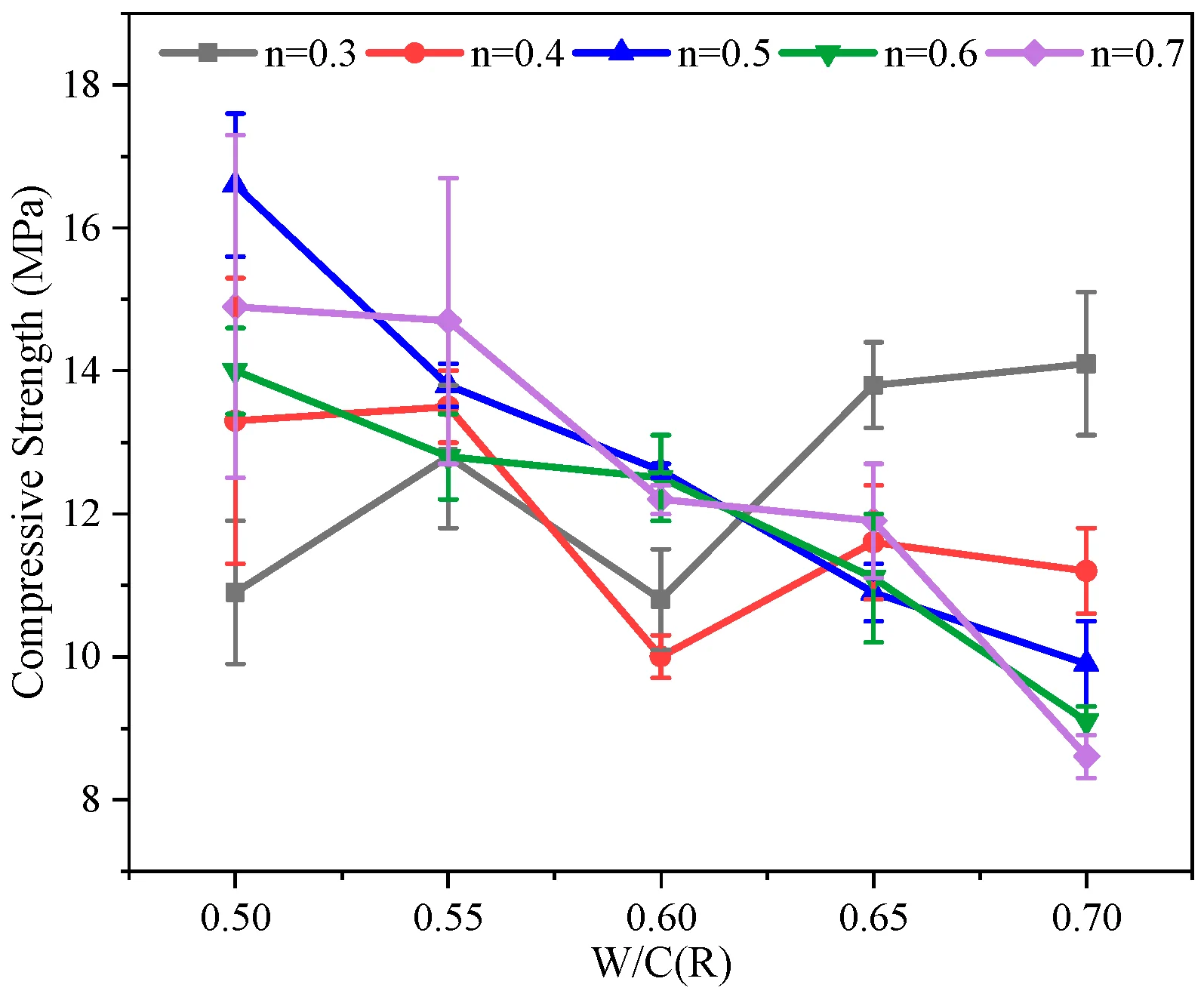
Compressive strength -W/C curve of gangue-based concrete with different grading parameters.

Figure 9 presents the relationship between compressive strength and the water–cement ratio of gangue-based concrete with different grading parameters after three days curing. Statistical parameters of compressive strength of gangue-based concrete with different grading parameters are presented in Table 4. For gangue-based concrete with grading parameters of 0.3 and 0.4, the uniaxial compressive strength fluctuates with the water–cement ratio. When the grading parameter is lower, the proportion of aggregates with a particle size under 2.5 mm exceeds 50% as shown in Figure 7. Achieving uniform mixing of the concrete becomes difficult at lower water–cement ratios, leading to agglomeration within the concrete. When the grading parameter is higher than 0.4 (n = 0.5, 0.6, 0.7), the uniaxial compressive strength gradually decreases with the increase in the water–cement ratio. For n = 0.5, the uniaxial compressive strength decreases linearly with the water–cement ratio. For the grading parameter of 0.6, when the water–cement ratio is less than 0.6, its uniaxial compressive strength decreases slowly with the water–cement ratio. However, when the water–cement ratio exceeds 0.6, its uniaxial compressive strength decreases rapidly. When the aggregate grading parameter increases to 0.7, the uniaxial compressive strength does not change significantly when the water–cement ratio is less than 0.55. But when the water–cement ratio exceeds 0.55, the uniaxial compressive strength decreases rapidly. The decrease in uniaxial compressive strength is mainly caused by changes in the concrete’s microstructure and compactness with increasing water–cement ratio. Excessively high water–cement ratios result in a large amount of free water adsorbed around the aggregates, leading to the loose microstructure of ITZ.

Based on the influence of aggregate grading and water–cement ratio on the workability and uniaxial compressive strength of gangue-based concrete, this study selects a grading parameter of 0.6 and a water–cement ratio of 0.6 as the reasonable mix proportion. Subsequent relevant tests cast gangue-based concrete specimens using this mix proportion.

## 5. Effect of Gangue Lithology on Mechanical Properties of Concrete

Six gangue-based concrete mixtures were designed to analyze the influence of gangue lithology and mixing on the mechanical behavior of gangue-based concretes. The aggregate grading parameter for the six mixtures was 0.6, and the water–cement ratio was 0.6. The specific mix proportions are shown in Table 3.

### 5.1. Compressive Stress–Strain Curve

**Figure 10:**
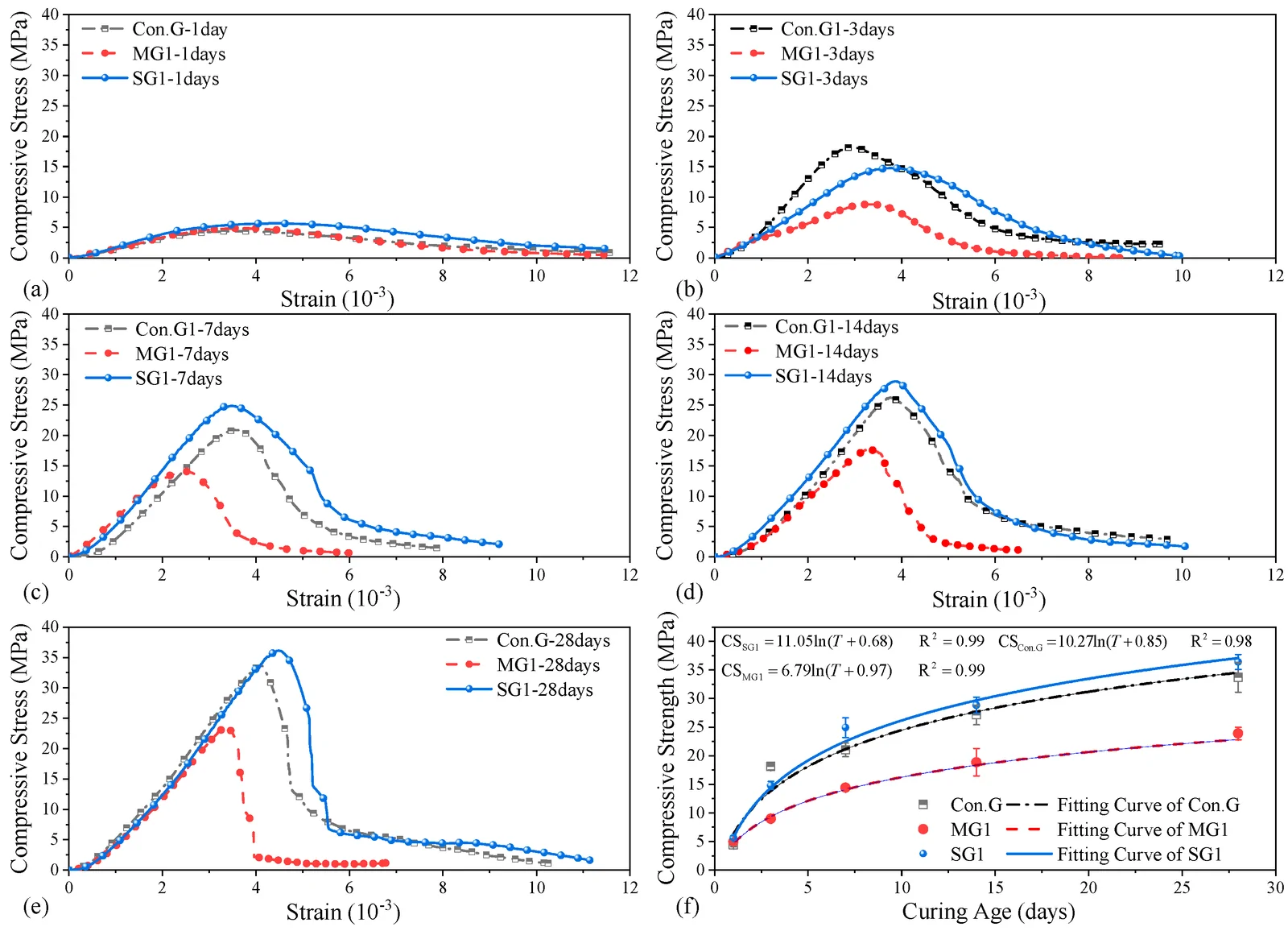
Compressive stress–strain curves of single lithology gangue-based concretes at different curing ages.

Figure 10 shows the compressive stress–strain curves of single lithology gangues-based concrete at different curing ages. The uniaxial compressive strength of single-lithology gangue-based concrete increases with curing time, exhibiting distinct brittle failure characteristics. After 1-day curing, the stress–strain curves of gangue-based concretes with different lithologies are similar. During the initial stages of curing, the hydration reaction is relatively low, and the uniaxial compressive failure of the concrete is mainly caused by the rupture of the concrete matrix. At this stage, the concrete displays obvious plastic failure characteristics. When the curing time reaches 3 days or more, significant differences appear in the stress–strain curves of gangue-based concretes with different lithologies, and the brittleness of the concrete increases. The stress–strain curves of sandstone gangue-based concrete with increasing curing age are consistent with those of the control group (limestone aggregate). And its uniaxial compressive strength is higher than that of mudstone gangue-based concrete. With the increasing curing time, the hydration degree rises, enhancing the microstructural strength and thus improving its load-bearing capacity. When the concrete matrix attains a certain strength, the damage and failure of the concrete are primarily determined by the microstructural strength of ITZ. The ITZ of mudstone gangue-based concrete has a loose structure, resulting in crack propagation under relatively low loads.

The uniaxial compressive strengths of gangue-based concretes with different lithologies at various curing ages are shown in Figure 10f. The uniaxial compressive strength increases with the curing time, displaying a rapid gain during the first 7 days. During the initial curing period, the uniaxial compressive strengths of the mudstone gangue-based concrete and sandstone gangue-based concrete are similar to that of the control group. However, as the curing time increases, the compressive strength of sandstone gangue-based concrete surpasses that of mudstone gangue-based concrete. This is primarily attributed to the lower strength of mudstone gangue, which undergoes disintegration when exposed to water during curing, further reducing its strength. This leads to aggregate fracture and debonding between hydration products and aggregates, thereby destroying the main load-bearing structure of the concrete. In contrast, sandstone gangue and limestone exhibit higher strengths and good water stability, enabling them to withstand higher external loads. This is mainly because their primary minerals are quartz and calcite without abundant hydrophilic clay minerals. They possess stable grain-supported or interlocking crystalline microstructures. Water molecules cannot easily damage their mineral lattice, whereas mudstone rich in clay minerals suffers severe softening after water absorption.

As depicted in Figure 10f, an logarithmic relationship (Equation (2)) was detected between the compressive strength and curing time for considered gangue-based concretes. The coefficients of correlation can assess the reliability of the logarithmic interpolation. The coefficients of correlation are above 0.95. The logarithmic relationship highlighted the evolution in time of compressive strength during the hydration process.

CS = a ⋅ ln(*T* − *b*) (2)

where CS is the compressive strength of gangue-based concrete (MPa), *T* is the curing time of concrete (days), a and *b* are constants. For MG1, SG1 and Con.G, a and *b* are 6.79, 11.05, 10.27 and −0.97, −0.68, −0.85.

### 5.2. Effect of Gangue Lithology on Elastic Modulus

**Figure 11:**
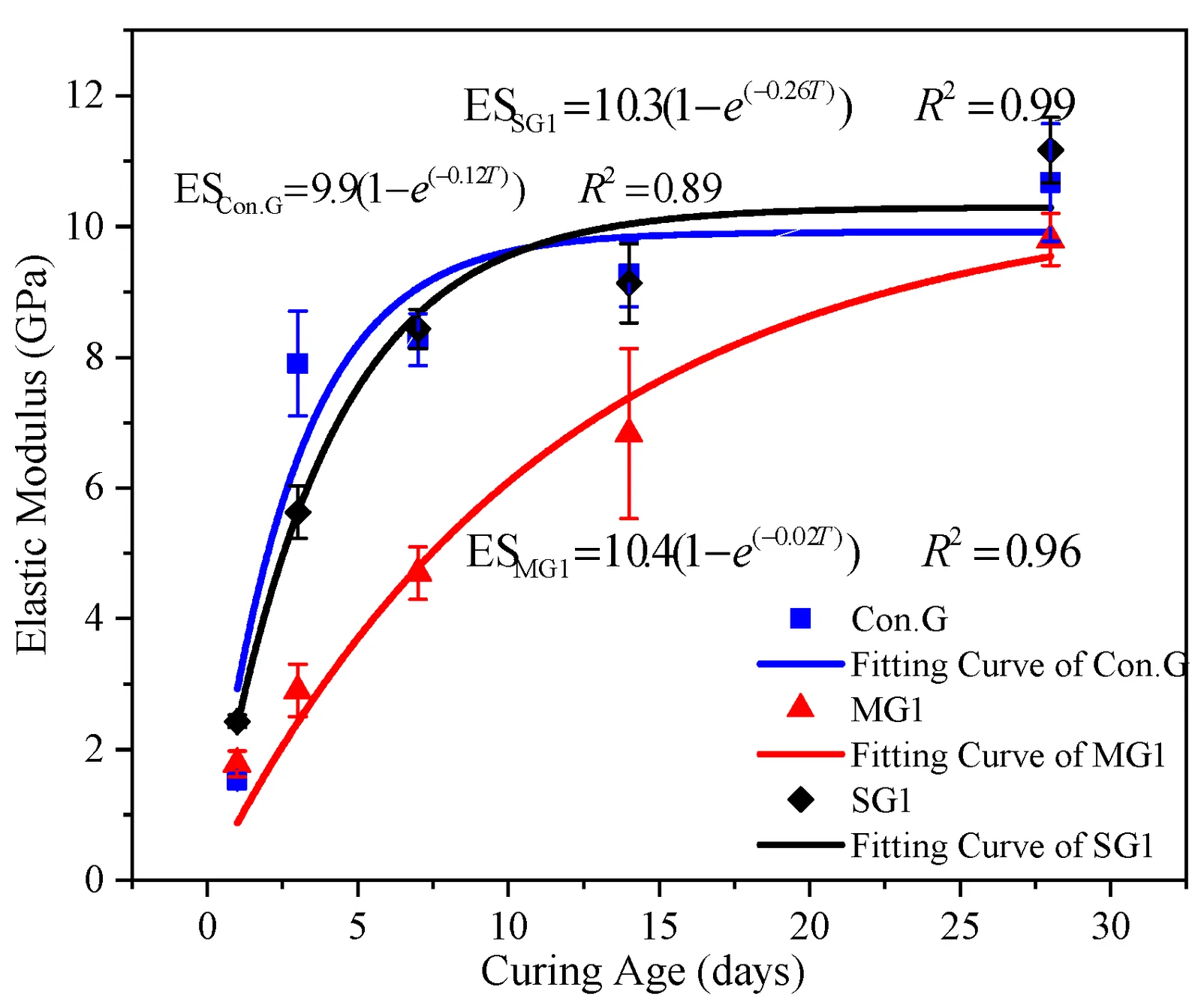
Elastic modulus with different curing ages of different groups.

Figure 11 shows the elastic modulus of gangue-based concretes with different coal gangue at different curing ages. The elastic modulus is defined as the secant modulus corresponding to 50% of the compressive strength on the compressive stress–strain curve. Within the 28-day curing period, the elastic modulus of sandstone based gangue concrete is comparable to that of the control group and higher than that of mudstone gangue-based concrete. After 1 day of curing, the elastic moduli of the three types of concrete are similar. During the initial stage, the degree of hydration reaction is relatively low, leading to plastic deformation of the concrete under low loading. At this stage, the elastic modulus of the concrete is primarily determined by the hydration reaction products. With increasing curing time, the elastic modulus of sandstone gangue-based concrete exceeds that of mudstone gangue-based concrete. Notably, when the curing age reaches 7 days, the elastic modulus of mudstone gangue-based concrete is only 50% of that of sandstone gangue-based concrete. It is related to the reduced stability and strength of mudstone gangue when exposed to water. Additionally, mudstone gangue has strong water absorption capabilities, and a large amount of free water is adsorbed on the surface of mudstone gangue during the initial curing stages, resulting in a relatively loose microstructure of hydration products in the interfacial transition zone near the gangue. Consequently, under external loads, microcracks initiate in the interfacial transition zone and propagate into the concrete matrix, ultimately leading to plastic deformation of the mudstone gangue-based concrete under relatively low loads. As the curing time increases, the elastic modulus of sandstone gangue-based concrete increases slowly and stabilizes after 15 days of curing. In contrast, the elastic modulus of mudstone gangue-based concrete gradually increases with the curing time. After 28 days of curing, the elastic modulus reaches 9.8 GPa, approaching that of sandstone gangue-based concrete. With increasing curing time, the degree of hydration reaction in the concrete continues to increase, enhancing the strength of the concrete matrix. When the strength of the concrete matrix exceeds that of the mudstone gangue, the matrix becomes the primary load-bearing structure and inhibits the propagation of cracks within the mudstone gangue.

As depicted in Figure 11, an exponential relationship (Equation (3)) is detected between the Elastic modulus and curing time for all considered gangue-based concretes. The coefficients of correlation are above 0.88. This exponential correlation characterizes the time-dependent evolution of elastic modulus throughout cement hydration. It may be used to predict the mechanical properties of gangue-based concrete.

EM = m(1 − *e*^(−*nT*)^) (3)

where EM is the compressive strength of gangue-based concrete (GPa), *T* is the curing time of concrete (days), m and *n* are constants. For MG1, SG1 and Con.G, m and *n* are 10.4, 10.3, 9.9 and 0.02, 0.26, 0.12.

### 5.3. Effects of Different Lithofacies Gangue Mixing Ratios on Mechanical Behaviors of Gangue-Based Concrete

**Figure 12:**
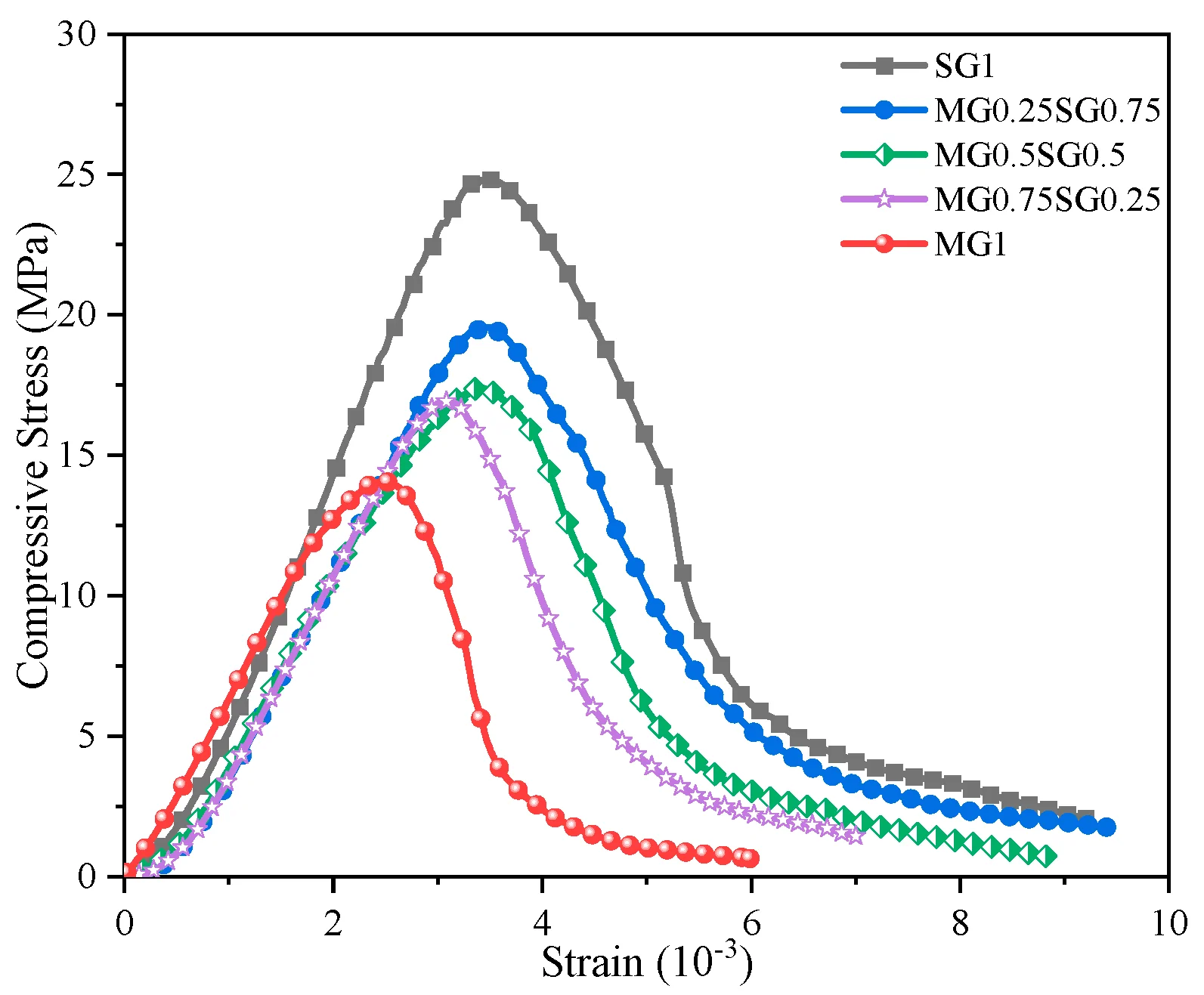
Stress–strain curve of the mixed gangue-based concrete.

Figure 12 presents the uniaxial compressive stress–strain curves of gangue-based concrete mixed with varying proportions of sandstone gangue and mudstone gangue at a 7-day curing age. With the increase in the mudstone gangue, a noticeable deterioration in the uniaxial compressive mechanical properties of the gangue-based concrete appears. When 25% (MG0.25SG0.75) and 50% (MG0.5SG0.5) of the sandstone gangue in the sandstone gangue-based concrete (SG1) are replaced by mudstone gangue, there are no significant changes in the uniaxial compressive stress–strain curves, except for the decrease in the compressive strength. However, when 75% of the sandstone gangue is replaced by mudstone gangue, not only does the uniaxial compressive strength of the concrete decrease, but the axial strain at the maximum uniaxial compressive stress also decreases. When the content of mudstone gangue in the concrete exceeds a certain limit (75%), the microstructure strength of the gangue-based concrete decreases, resulting in the overall failure of the concrete with relatively small deformations.

**Figure 13:**
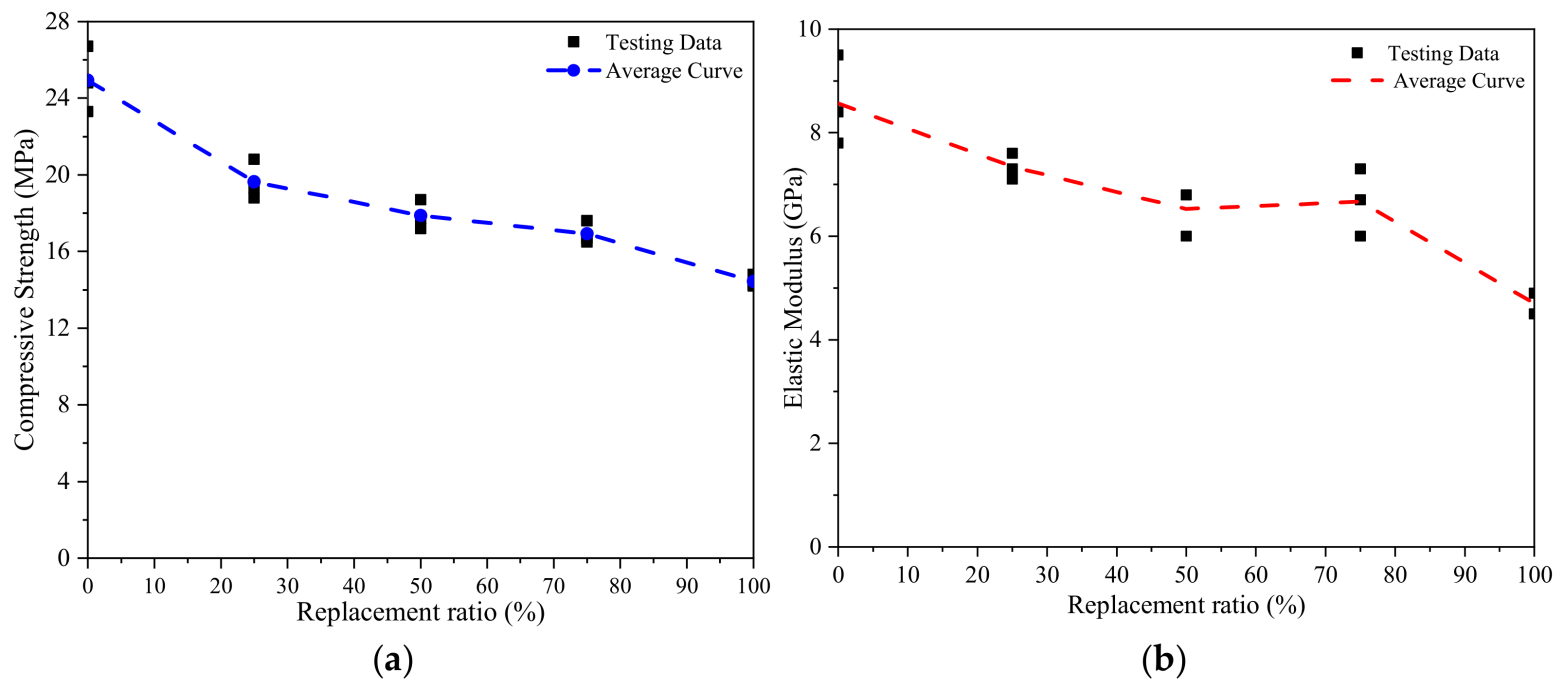
Compressive strength (**a**), and elastic modulus (**b**) of mixed gangue-based concrete.

Figure 13 presents the relationship between the uniaxial compressive strength and elastic modulus of mixed gangue-based concrete at a 7-day curing age. The uniaxial compressive strength of sandstone gangue-based concrete gradually decreases with the increase in mudstone gangue content. When 25% of the sandstone gangue is replaced by mudstone gangue, the uniaxial compressive strength of the concrete drops by 21.2%. It is mainly caused by the disintegration of mudstone gangue during hydration when exposed to water and the resulting porous ITZ. When the replacement rates are 50% and 75%, the uniaxial compressive strength of the gangue-based concrete decreases by 30.1% and 31.2%, respectively. The compressive strength experiences a sharp decrease (43.5%) when the replace ratio exceeds 75%. For engineering application, some sandstone coal gangue should be added when the mudstone coal gangue exceeds 75%.

The elastic modulus of sandstone gangue-based concrete gradually decreases with the increase in mudstone gangue content. For gangue-based concrete with mudstone gangue incorporation ratios of 25%, 50%, 75%, and 100%, the average elastic modulus decreases by 14.4%, 23.8%, 22.2%, and 45.2%, respectively. When all the sandstone gangue is replaced by mudstone gangue, the elastic modulus of the concrete drops sharply, suggesting that when the mudstone gangue content exceeds 75%, the gangue-based concrete’s ability to resist plastic deformation significantly decreases.

## 6. Effects of Gangue Lithology on Failure Modes and Microstructure of Gangue-Based Concrete

### 6.1. Effect of Gangue Lithology on the Failure Model

**Figure 14:**
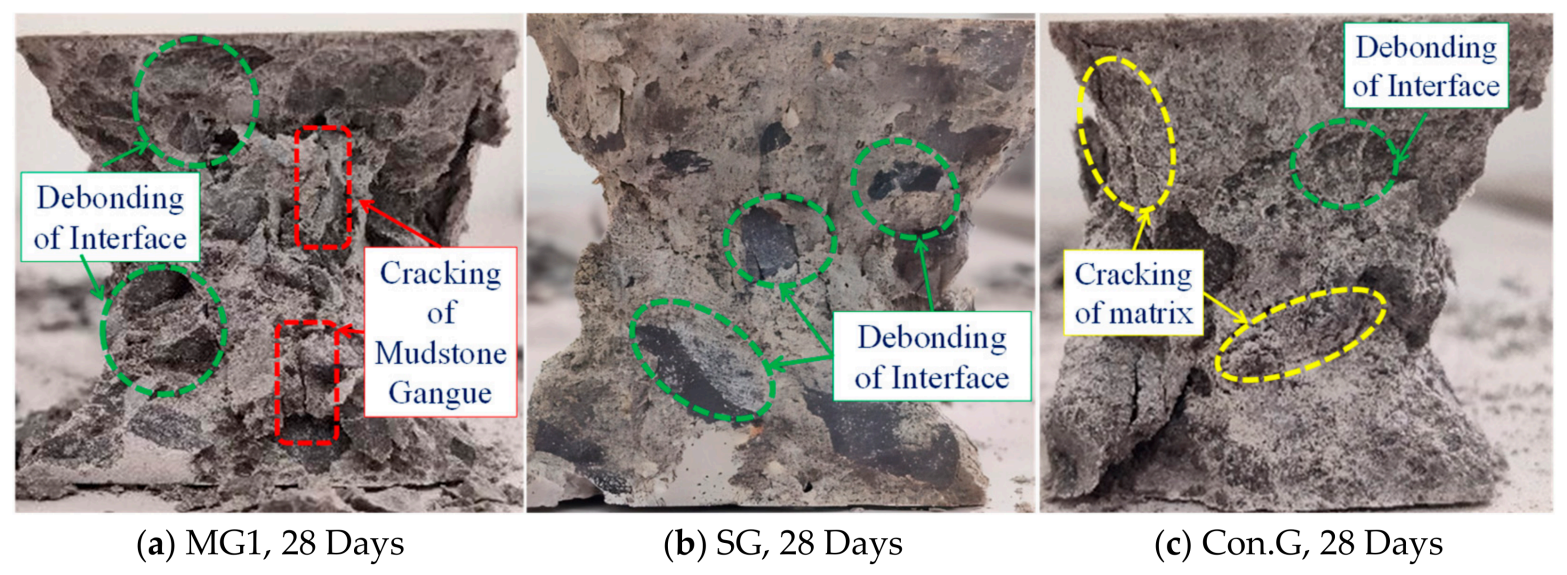
Failure models of gangue-based concrete: (**a**) mudstone gangue, (**b**) sandstone gangue, and (**c**) normal concrete.

Figure 14 presents the failure modes of gangue-based concrete after 28 days of curing. It can be observed that gangue lithology exerts an essential influence on the failure modes of gangue-based concrete. For concrete containing mudstone gangue, the main failure modes are the debonding of the interface between aggregates and the matrix, as well as the cracking of mudstone gangue. In contrast, concrete incorporating sandstone and limestone fails due to interfacial debonding and matrix cracking. These distinct failure modes are closely related to gangue lithology. From a microstructural perspective, microcracks initiate and propagate from the interfacial transition zone (ITZ). With the increase in loading, the microcracks extend to the matrix and aggregates, resulting in the collapse of the load-bearing microstructures. For concrete with mudstone aggregates, the ITZ exhibits a loose microstructure due to the strong water absorption characteristics of mudstone, leading to interfacial debonding at a relatively low loading level. Meanwhile, mudstone aggregates have a weak microcrack arresting effect owing to their lower strength. In contrast, sandstone gangue and limestone possess higher strength and good water stability, enabling them to withstand higher external loads. Under external loading, micro-damage propagates from the ITZ to the concrete matrix, ultimately leading to concrete failure characterized by interfacial debonding between gangue and matrix, as well as matrix cracking.

**Figure 15:**
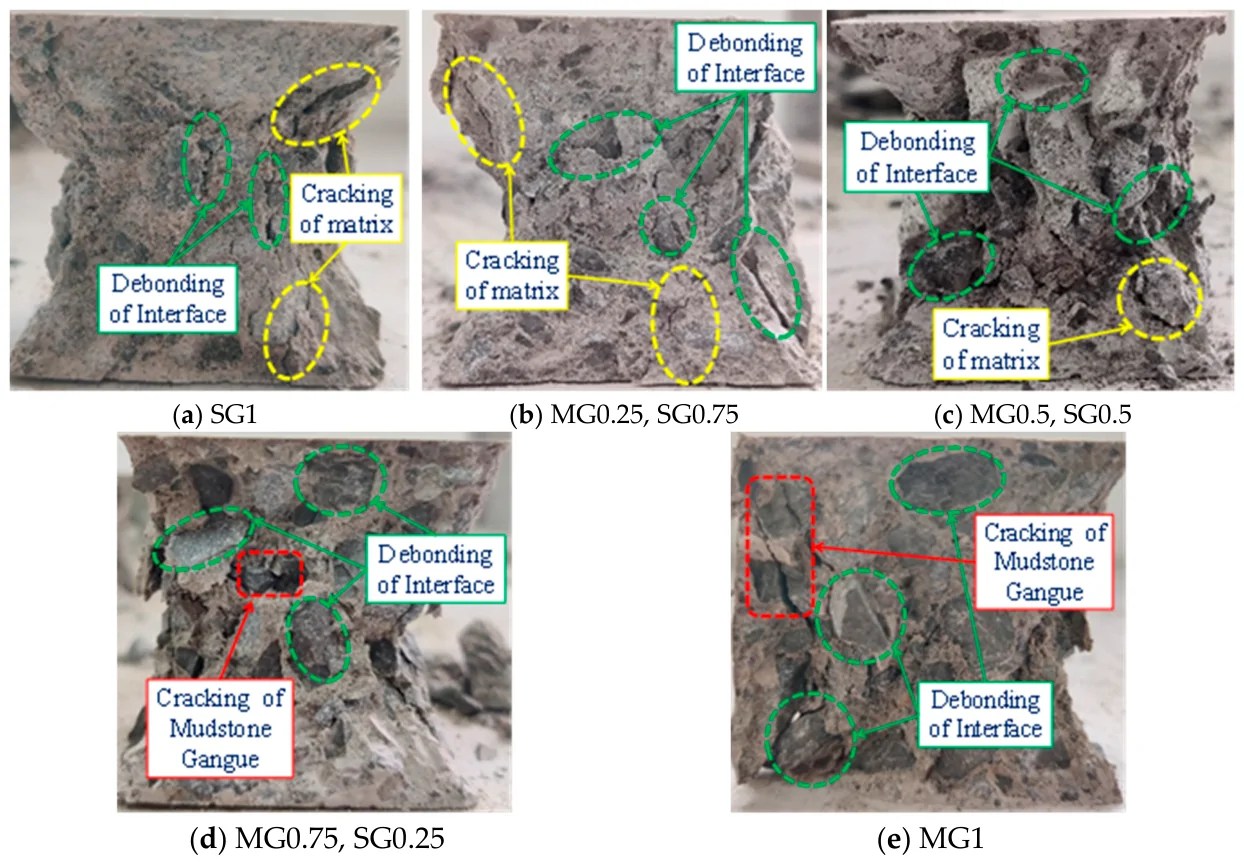
Failure modes of gangue-based concrete with different lithofacies mixing proportions.

Figure 15 illustrates the failure modes of gangue concrete with different lithofacies mixing ratios. For coal gangue-based concrete with a high sandstone content, the failure is dominated by matrix cracking and ITZ failure. With an increasing proportion of mudstone, however, the fracture of gangue aggregates gradually becomes prominent and replaces matrix cracking as one of the primary failure modes. This indicates that mudstone gangue aggregates act as one of the crack initiation sources in concrete, whereas sandstone coarse aggregates can provide a relatively higher bearing capacity.

### 6.2. Effect of Gangue Lithology on the Microstructure

**Figure 16:**
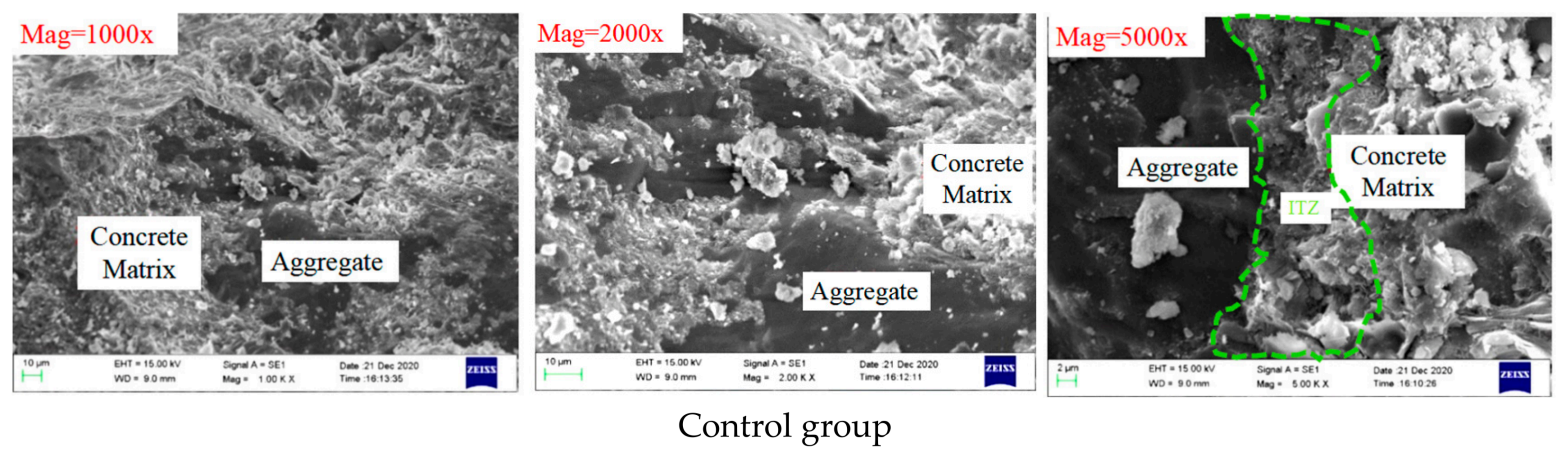

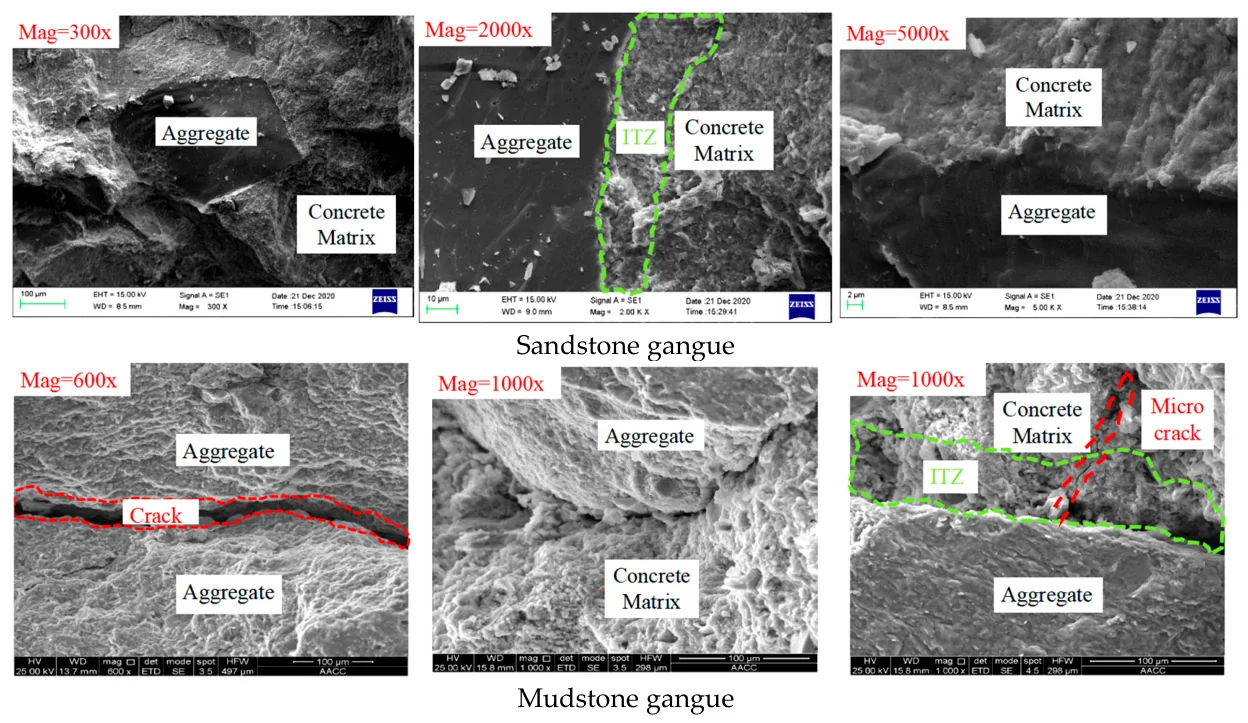
Microstructures of gangue-based concretes.

Figure 16 presents the SEM images of the ITZ of gangue-based concrete after 28 days of curing. The ITZ is a relatively weak part of the concrete microstructure, and its morphological characteristics significantly influence the mechanical properties of the concrete [37,38,39]. For the control group, the majority of limestone aggregates are covered by a dense concrete matrix. The ITZ is tightly structured, resulting in high strength. The microstructure of the ITZ in sandstone gangue concrete is similar to that of the control group. However, for mudstone gangue-based concrete, the bonding between the aggregates and the concrete matrix is relatively weak, and microscopic cracks appear between the gangue and hydration products. The ITZ microstructure is porous and has certain micro-cracks. Additionally, in mudstone gangue-based concrete, some mudstone gangue cracks after water absorption. Under loading, the micro-cracks within the ITZ propagate and coalesce, further damaging the microstructure of the ITZ. The microstructural damage in the ITZ prevents stress transfer between the aggregates and the matrix, leading to increased stress concentration within the ITZ. This, in turn, prompts cracks to extend from the ITZ into the concrete matrix.

The differences in the microstructure of the ITZ between mudstone gangue-based concrete and sandstone gangue-based concrete are primarily related to the cement hydration reactions. Immersion tests have shown that sandstone has better water stability and lower water absorption compared to mudstone. Therefore, there is less free water on the surface of sandstone gangue, leading to a denser microstructure of C-S-H and Ca(OH)_2_. In contrast, mudstone gangue adsorbs a large amount of free water on its surface. The cement particles around the mudstone gangue undergo hydration reactions with a higher water–cement ratio, leading to the formation of hydrated products with a porous and loose microstructure in ITZ. Additionally, the water-induced disintegration of mudstone gangue results in delamination at the interface between the aggregates and the cement paste, causing more severe damage to the interface.

**Figure 17:**
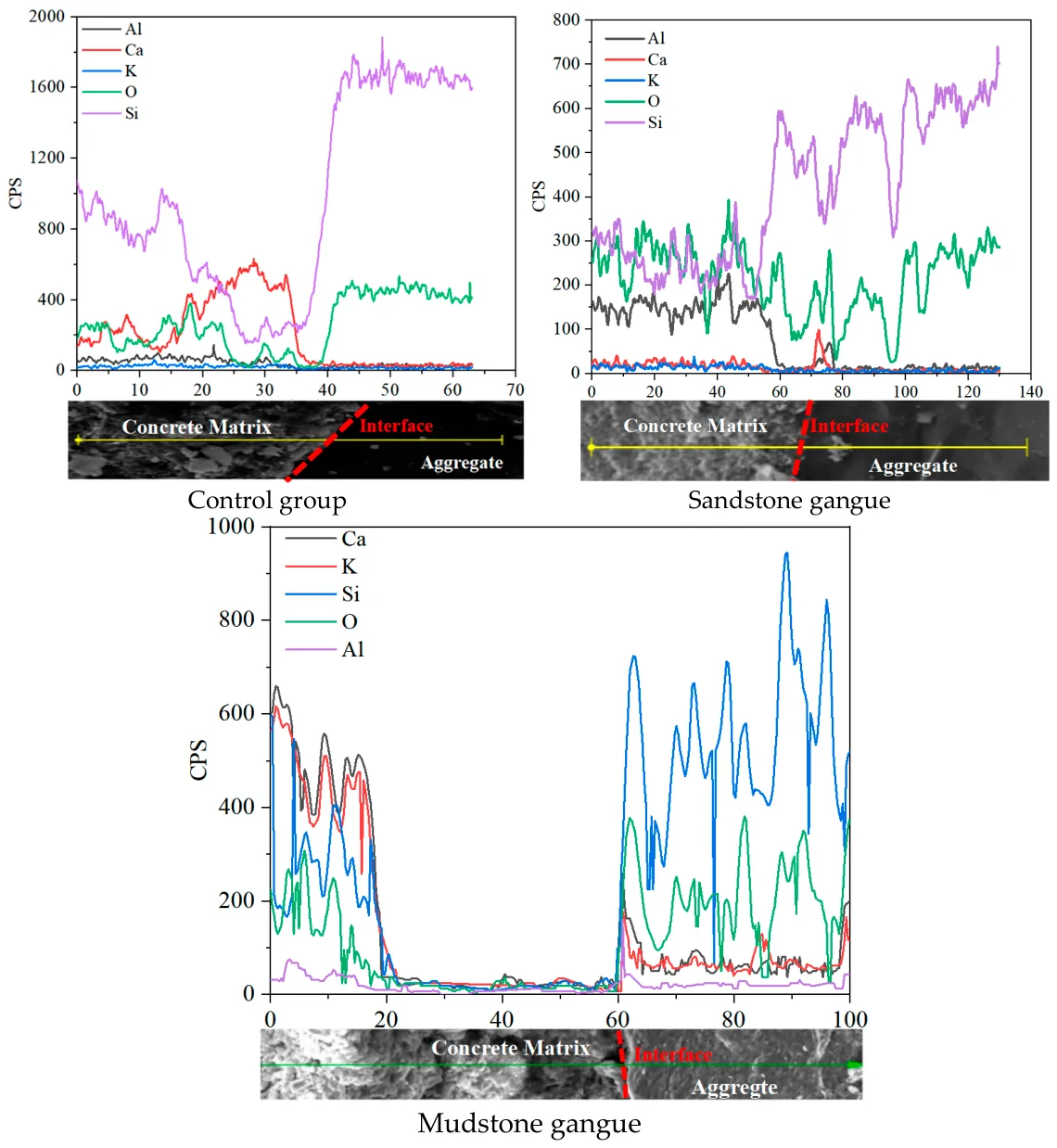
Chemical element distribution in the IZT.

Using the Energy Dispersive Spectrometer (EDS) associated with the Scanning Electron Microscope (SEM), the migration and aggregation of chemical elements in the interfacial transition zone (ITZ) were analyzed. The specific results are shown in Figure 17. The sandstone gangue and limestone contain high levels of silicon and oxygen, while the mudstone gangue has higher concentrations of elements such as Si, O, and Al. From the aggregate towards the concrete matrix, there is a sharp decrease in the silicon (Si) content in the sandstone within the ITZ, while the aluminum (Al) content in the concrete matrix shows a significant drop. In terms of the main hydrated product components, the concrete matrix is rich in calcium (Ca) phase, but the Ca content in the concrete matrix is relatively low in the chemical element distribution map. It is primarily due to the reactivity of the sandstone gangue, which generates a significant amount of C-S-H, covering the surface of the concrete matrix during hydration. Similarly, there is a notable decrease in silicon and oxygen elements at the interface in limestone, but there is a significant increase in Ca elements. This is mainly because the cement particles around the limestone undergo incomplete hydration reactions, producing a large amount of Ca(OH)_2_. In the direction from the mudstone aggregate to the matrix, the contents of Si, O, and Al in the aggregate undergo a sharp decrease at the interface, while the contents of Ca, Si, O, and Al in the matrix also show a significant drop at the interface. The mudstone gangue adsorbs a large amount of free water on its surface, leading to complete hydration reactions of the cement particles around the gangue, generating a large number of hydrated products such as C-S-H.

## 7. Discussion

**Figure 18:**
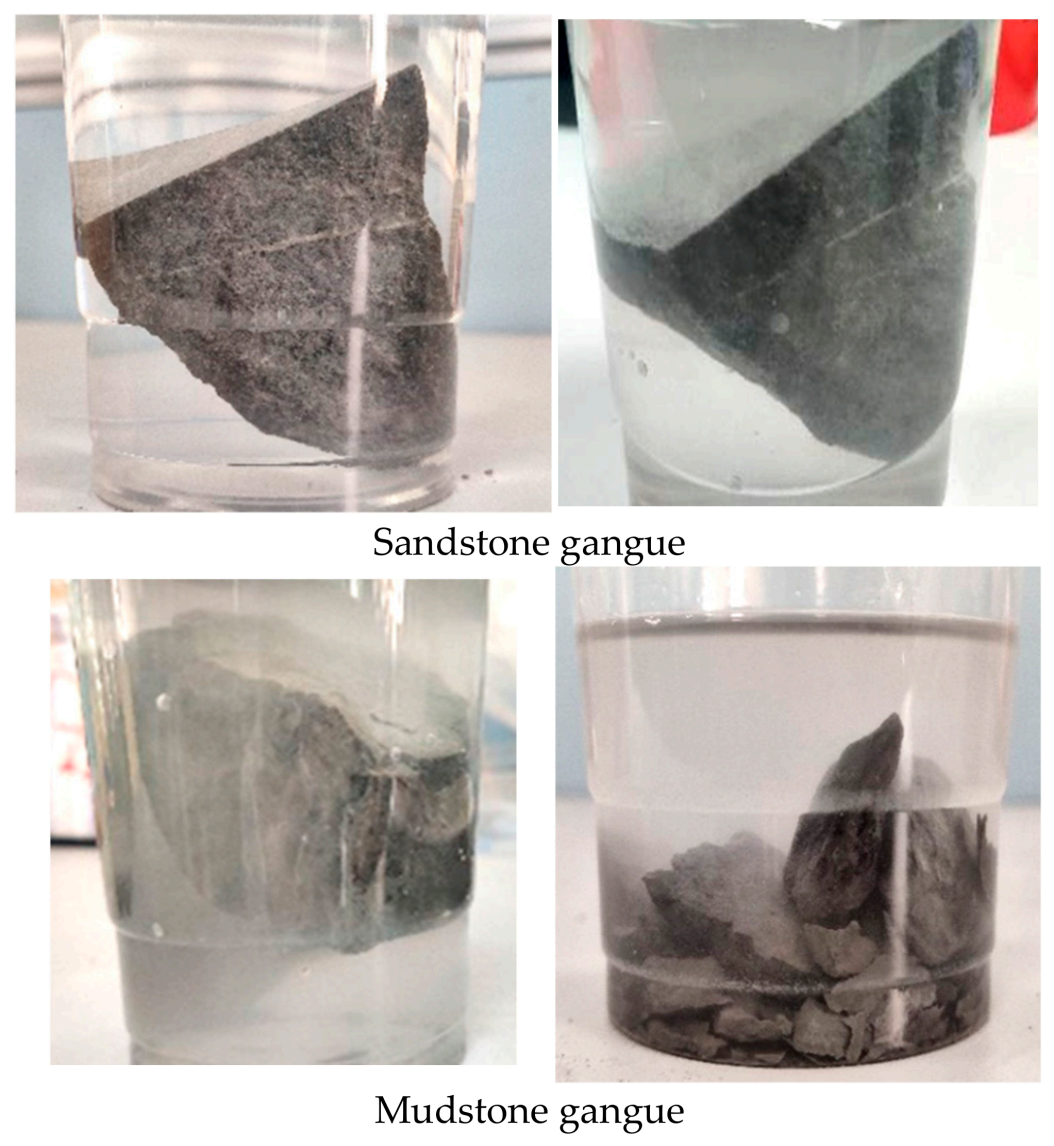
Disintegration characteristics of coal gangue soaked in water.

The results of this investigation indicate that with an increase in argillaceous gangue content, the strength of gangue-based concrete decreases significantly and its damage and fracture mode is altered. This is directly related to the strong water absorption and water-induced disintegration of coarse mudstone aggregates. Coal gangue that is prone to disintegration upon contact with water can easily break down during the concrete mixing and hydration processes, losing its role as an aggregate to support loads [40]. Additionally, the disintegration of gangue aggregates in water creates numerous micro-cracks in the concrete, leading to its failure under relatively low external loads. The results of disintegration tests for sandstone gangue and mudstone gangue are shown in Figure 18. After being immersed in water for one week, the sandstone gangue did not change significantly, indicating the good stability when exposed to water. However, mudstone gangue completely disintegrated into small fragments.

The disintegration characteristic of mudstone gangue is related to the micro cracks and voids within it, as well as its mineral composition. Mudstone contains abundant clay minerals such as kaolinite and illite. Water molecules readily penetrate into the interlayers of clay mineral crystals due to their large specific surface area, inducing hydration expansion and weakening the bonding force between particles [24,41,42]. A large number of bedding planes and microcracks are developed inside mudstone. Water further infiltrates along these cracks and generates pore water pressure, eventually resulting in softening, swelling and disintegration of mudstone [43,44]. In contrast, sandstone is composed of high-strength quartz grains with a grain interlocking supporting structure and barely contains strongly hydrophilic clay minerals. Water molecules cannot invade the mineral lattice but merely fill intergranular pores, so the mineral matrix and inter-particle interlocking structure remain intact, and sandstone maintains structural stability with slight strength reduction upon water immersion. For concrete including mudstone coal gangue aggregate, the mudstone aggregate absorbs water and undergoes hydration expansion during concrete mixing. This behavior induces internal damage within the aggregate and degrades the interfacial transition zone (ITZ) between aggregate and cement paste. Cracks tend to initiate and propagate preferentially inside the weak aggregate or along the ITZ under external load, thereby reducing the macroscopic strength of concrete.

The presence of argillaceous gangue constitutes the primary cause for the strength reduction in gangue-based concrete. As the proportion of argillaceous gangue increases, the compressive strength of gangue-based concrete exhibits an approximately linear decline. When gangue containing argillaceous components is employed as aggregate, the content of argillaceous gangue must be strictly controlled and determined. If the argillaceous gangue content exceeds a specified threshold (75%), non-argillaceous aggregates such as granite should be incorporated into the original aggregate system.

## 8. Limitations and Future Prospects

This study systematically reveals the effects of gangue lithology and aggregate grading parameters on the mechanical properties and failure modes of coal gangue aggregate concrete, and proposes an optimized mixture design method. Nevertheless, several limitations remain. The present work only focuses on static compressive behavior under standard curing conditions. Long-term durability properties including drying shrinkage, creep, freeze–thaw resistance and sulfate attack resistance, as well as dynamic mechanical performance under fatigue or impact loading, remain unexplored. The influence of argillaceous gangue on cement hydration and interfacial transition zone (ITZ) microstructure is only qualitatively analyzed based on SEM observations, lacking quantitative characterization of hydration kinetics and molecular-scale water–clay mineral interactions.

In response to the above limitations, core fundamental research directions are proposed for future work. First, systematic investigations will be carried out on the long-term durability and dynamic mechanical properties of high mudstone-content coal gangue concrete, to clarify the deterioration law and failure mechanism under various service environments and establish a full-cycle performance database. Second, advanced multi-scale characterization techniques including isothermal calorimetry, FTIR and XRD will be adopted to quantitatively reveal the hydration kinetics of gangue-blended cement systems and the modification mechanism of argillaceous gangue on the composition and microstructure of ITZ hydration products.

## 9. Conclusions

This study experimentally investigated how gangue lithology affects the mechanical properties and mix proportion optimization of gangue-based concrete. The main conclusions are summarized below:

(1) The workability of gangue-based concrete improves as the aggregate grading parameter increases, though this improvement becomes minor beyond 0.6. For grading parameters above 0.4, the uniaxial compressive strength decreases gradually with an increasing water–cement ratio. Considering the coupled effects of aggregate grading and the water–cement ratio on both workability and compressive strength, the optimal mix proportions are determined to be a grading parameter of 0.6 and a water–cement ratio of 0.6.
(2) Mudstone gangue incorporation exerts detrimental impacts on the mechanical performance of gangue-based concrete. For mudstone gangue-based concrete, the elastic modulus exhibits an exponential growth trend with curing age. In hybrid concrete blended with sandstone and mudstone gangues, both the uniaxial compressive strength and elastic modulus gradually degrade as the proportion of mudstone gangue rises. This deterioration becomes particularly prominent once the mudstone gangue replacement fraction surpasses 75%. From an engineering standpoint, incorporating supplementary sandstone gangue is recommended for mixtures where the mudstone content exceeds the 75% threshold to mitigate severe mechanical degradation.
(3) Mudstone gangue incorporation alters the failure modes of coal gangue aggregate concrete. As the mudstone gangue proportion increases from 25% to 100%, the dominant failure patterns under uniaxial compression shift from aggregate–matrix interfacial debonding and matrix cracking to a combined failure of interfacial debonding, aggregate rupture, and matrix fracture. Microstructural analysis indicates that the ITZ of mudstone gangue concrete contains substantial pores and visible microcracks, whereas sandstone gangue concrete possesses a dense, well-integrated ITZ microstructure.

## Figures and Tables

**Table 1:** Testing groups for optimizing aggregate gradation and water–cement ratio.

	W/C (R)	0.5	0.55	0.6	0.65	0.7
Grading (n)						
0.3		n0.3R0.5	n0.3R0.55	n0.3R0.6	n0.3R0.65	n0.3R0.7
0.4		n0.4R0.5	n0.4R0.55	n0.4R0.6	n0.4R0.65	n0.4R0.7
0.5		n0.5R0.5	n0.5R0.55	n0.5R0.6	n0.5R0.65	n0.5R0.7
0.6		n0.6R0.5	n0.6R0.55	n0.6R0.6	n0.6R0.65	n0.6R0.7
0.7		n0.7R0.5	n0.7R0.55	n0.7R0.6	n0.7R0.65	n0.7R0.7

**Table 2:** Concrete mixtures with different water–cement ratios.

W/C	Aggregate (Kg/m^3^)	Cement (Kg/m^3^)	Water (Kg/m^3^)
0.5	1640	410	205
0.55	1640	410	225.5
0.60	1640	410	246
0.65	1640	410	266.5
0.7	1640	410	287

**Table 3:** Concrete mixtures with different gangues.

Mix ID	Aggregate (Kg/m^3^)				Cement (Kg/m^3^)	Water (Kg/m^3^)	W/C
	MG	SG	LS	RS			
Con.G	-	-	820	820	410	246	0.6
MG1	1640	-	-	-			
SG1	-	1640	-	-			
MG0.25SG0.75	410	1230	-	-			
MG0.5SG0.5	820	820	-	-			
MG0.75SG0.25	1230	410	-	-			

**Table 4:** Statistical parameters of compressive strength of gangue-based concrete with different grading parameters.

	W/C(R)	0.5			0.55			0.6			0.65			0.7		
Grading (n)		M	SD	Va.	M	SD	Va.	M	SD	Va.	M	SD	Va.	M	SD	Va.
0.3		10.9	1.01	1.03	12.8	0.92	0.84	10.8	0.65	0.42	13.8	0.56	0.31	14.1	1.01	1.11
0.4		13.2	2.05	4.21	13.5	0.46	0.21	10.0	0.31	0.09	11.6	0.76	0.58	11.2	0.59	0.34
0.5		16.6	1.01	1.02	13.8	0.31	0.09	12.6	0.15	0.02	10.9	0.38	0.14	9.9	0.61	0.37
0.6		14.0	0.60	0.36	12.8	0.61	0.37	12.5	0.61	0.37	11.1	0.95	0.91	9.1	0.21	0.04
0.7		14.9	2.40	5.77	14.7	2.00	4.01	12.2	0.17	0.03	11.9	0.81	0.66	8.6	0.30	0.09

M denotes the mean value in MPa; SD represents the standard deviation in MPa; Va. stands for the variance in MPa^2^.

## Data Availability

The original contributions presented in this study are included in the article. Further inquiries can be directed to the corresponding author.
